# Mortality and survival of tuberculosis coinfected patients living with AIDS in São Paulo, Brazil: a 12-year cohort study

**DOI:** 10.1186/s12879-022-07232-6

**Published:** 2022-03-05

**Authors:** Mariza Vono Tancredi, Sumire Sakabe, Eliseu Alves Waldman

**Affiliations:** 1 State Department of Health, STD/AIDS Referral and Training Center, R. Santa Cruz, 81, São Paulo, CEP: 04121-000 Brazil; 2 Department of Epidemiology, School of Public Health, University of São Paulo, Av. Dr. Arnaldo 715, Cerqueira César, São Paulo, SP CEP: 01246-904 Brazil

**Keywords:** AIDS, Survival analysis, Tuberculosis–HIV co-infection, AIDS mortality

## Abstract

**Background:**

TB is still one of the leading causes of death among HIV patients. This study evaluates the effect of TB on the mortality rate, survival time, and predictors of survival in patients with AIDS living in different areas in São Paulo State (SPS).

**Methods:**

Retrospective cohort of adolescents and adults with AIDS, diagnosed between 2003 and 2007 and followed-up until 2014. Data were obtained from the Brazilian Ministry of Health. Mortality rates were estimated by person-years. Survival analysis used the date of diagnosis as the reference for the construction of Kaplan–Meier curves. The Cox model was used for the investigation of survival-associated factors.

**Results:**

A total of 35,515 patients were included, of whom 63.0% were male; 64.7% at the age group of 30 to 49 years, 64.4% were white, 12.9% co-infected with TB, and 37.6% had CD4 count above 200 cells/mm^3^ at diagnosis of AIDS. The 12-year survival probabilities were 74.1% and 55.7% among patients without and with TB co-infection, respectively. After adjustment for sex, age and year of diagnosis, the following exposures were independently associated with lower survival: residing in municipalities of the Interior (Hazard ratio (HR) = 1.43) and Coastal Area (HR = 1.9); illiteracy (HR = 2.61); being co-infected with TB (HR = 1.70); CD4 count below 200 cells/mm^3^ at AIDS diagnosis (HR = 2.31); viral load above 500 copies/ml at AIDS diagnosis (HR = 1.99); HAART1 regimen (one non-nucleoside reverse transcriptase inhibitor or boosted old protease inhibitors) (HR = 1.94).

**Conclusion:**

The impact of TB on survival of AIDS was heterogeneous, and affected by age, years of formal education, early AIDS diagnosis, and proper ARV treatment. These factors may not fully explain the different survival outcomes in each of the four regions within the same state. These results may subsidize focused interventions and public health policies conveying specific needs in each of the areas.

## Background

Until covid-19 pandemic, tuberculosis (TB) was the leading cause of death from a single infectious agent worldwide, accounting for one-third of all deaths among people living with HIV/AIDS. This disease is therefore an important factor associated with lower survival in this group of patients [1, 2]. The introduction of highly active antiretroviral therapy (HAART) has led to a reduction in the incidence of TB among patients with AIDS, regardless of CD4+ lymphocyte count at the start of treatment [3]. Furthermore, the combined use of antiretroviral therapy (ARV) and isoniazid preventive therapy can reduce the risk of TB by up to 76% in patients with AIDS [4].

In Brazil, the Unified Health System (SUS in the Portuguese acronym) provides universal and free of charge health care and that includes diagnosis and treatment for TB and HIV/AIDS. In São Paulo state, TB diagnosis and treatment is provided by the primary health care, where HIV diagnosis is also offered, often with rapid tests. While HIV clinics are less numerous than primary health units, both are spread around cities and towns to provide access. The introduction of HAART in 1996 led to a substantial decline in mortality and an increase in the survival of patients living with HIV/AIDS [5–7]. In Brazil, the use of HAART resulted in a significant drop in the incidence of opportunistic infections, AIDS-defining cancers, and TB in patients with AIDS. However, this decrease did not occur homogenously across different regions or distinct social strata [2, 8], reflecting regional disparities in the prevalence of AIDS, TB, and TB–HIV co-infection [9, 10].

The burdens of AIDS and TB in São Paulo are high. In 2014, the last year of this study follow-up, new 7137 and 16,477 AIDS and TB cases were reported and represented 15 and 24% of all AIDS and TB new cases in Brazil [10]. The prevalence of TB–HIV co-infection in São Paulo state has decreased from 11.8% in 2007, to 9.1% in 2014, and continues to decline, having reached 7.2% in 2019 [10]. Despite of the decrease on co-infection rates, the impact of the dual infection remains high, with strong impact on mortality. A cohort of TB patients in São Paulo state between 2010 and 2015, showed 38.9% 5-year mortality rate after the first TB episode for HIV infected patients and 11.5% for the HIV negative group [11].

Considering the high burden of AIDS and TB in São Paulo and the paucity of publications on the importance of TB–HIV co-infection in the state, we conducted this study to estimate mortality rates and the median survival time in AIDS patients diagnosed between 2003 and 2007, and followed-up until 2014 with and without TB. In addition, we investigated the importance of TB co-infection as a predictor of survival in a cohort of patients with AIDS living in the four different areas of the state, each one with different incidences of AIDS and TB.

## Methods

This is a retrospective cohort study that evaluated all patients with AIDS aged 13 years or older reported to the São Paulo epidemiological surveillance system, who were diagnosed with AIDS between 2003 and 2007 and followed up until December 31, 2014. This study is part of a survey that covers all cases of AIDS reported in Brazil over the same period, which compose the BIAIDS-Brasil Cohort, a survival analysis of AIDS in Brazil [12].

### Settings

The study area was São Paulo state, with a population of 44 million inhabitants in 2014 [13]. In that year, with 0.783, São Paulo was the Brazilian state with the highest Human Developing Index (HDI) score. Among its municipalities, the HDI ranged between 0.862 and 0.639. Wealth distribution inequality, measured by Gini index also showed a large range within the state, from 0.3339 to 0.6555, mean 0.5768 [14]. These indicators point to a degree of income inequality; and the same is observed for the burden of HIV/AIDS and TB within São Paulo. We evaluated each of the four regions in which São Paulo state is divided by the State TB Program, aggregating municipalities with similar epidemiological characteristics [9]. Approximate population size and TB and AIDS incidence (new cases/100,000 inhabitants) for each of the four areas in 2014 were: (i) São Paulo City (SPC, state capital) 12 million inhabitants, TB and AIDS incidence rates 46.3 and 25.2/100,000 inhabitants per year, respectively; (ii) Greater São Paulo Region (GSPR) 9 million people, incidence rates of 30.9 (TB) and 14.1 (AIDS); (iii) São Paulo State Coastal Area (SPCA) 2 million inhabitants and incidence rates of 78.9 (TB) and 23.1 (AIDS), and (iv) Interior São Paulo State (ISPS) 21 million inhabitants and incidence rates of 21.9 (TB) and 15.1(AIDS) [9, 10]. HIV testing rate in TB cases in 2014 was 89.5% for the whole state, with the capital having the lowest testing rate, 85.6% and the GSPR with the best result, 93.1%. In the state, 2014 TB death rate per 100,000 inhabitants was 1.7, being lowest in ISPS, 1.1, and highest, 3.3, in SPCA [9].

### Population definitions

New cases of AIDS of both sexes, aged 13 or older, living in São Paulo state and reported to the AIDS surveillance system were included in the study population. Patients who died within 30 days of being diagnosed with AIDS were excluded. HIV infected individuals without AIDS and those registered with and monitored for less than 30 days by the Sistema de Controle de Exames Laboratoriais (SISCEL—Laboratory Test Control System) and/or by the Sistema de Controle Logístico de Medicamentos (SICLOM—Logistics Control System of Medicines) were also excluded.

An adult case of AIDS [15] was defined as any individual aged 13 or older with HIV infection confirmed by two sequential tests, first ELISA followed by Western blot or indirect immunofluorescence or PCR as confirmatory test, who had at least one AIDS-defining disease and/or CD4+ T lymphocyte count below 350 cells/mm^3^, regardless other causes of immunodeficiency. Death from AIDS was defined as any case whose underlying cause of death was AIDS, reported as B20 to B24 of the 10th version of the International Classification of Diseases (ICD-10) [16, 17].

TB in HIV-positive adults was defined when the diagnosis of AIDS was confirmed by the Rio de Janeiro/Caracas criterion in the presence of disseminated/extrapulmonary/non-cavitary TB or cavitary pulmonary TB or not specified. The cases included in the study who had TB at the diagnosis of AIDS or during follow-up were defined as TB–HIV co-infection [18].

### Antiretrovirals (ARV)

Treatment regimens were classified as: (i) pre-HAART (nucleoside analogue reverse transcriptase inhibitor only); (ii) HAART1 (containing one non-nucleoside reverse transcriptase inhibitor or boosted old protease inhibitors); (iii) HAART2 (containing what was considered, at the study period, third-line ARV and could only be prescribed if guided by genotypic test: darunavir, tipranavir, raltegravir, dolutegravir, etravirine, enfuvirtide, or maraviroc), for a minimum period of 30 consecutive days [19]. A fourth group of patients included the ones who did not use ARV during the follow-up period.

### Data extraction

The following four Brazilian Ministry of Health databases were used as data sources: (i) Sistema de Informação de Agravos de Notificação (SINAN—Notifiable Diseases Information System); (ii) SISCEL; (iii) SICLOM; (iv) Sistema de Informação sobre Mortalidade (SIM—Mortality Information System).

The following variables were studied: sociodemographic characteristics (age on the day of diagnosis of AIDS, sex, education, race/skin color, region of residence); exposure category, diagnosis and death (calendar year of diagnosis, CD4+ T lymphocyte count, viral load, underlying cause of death, calendar year of death, calendar year of censoring, interval between diagnosis and death); clinical characteristics (opportunistic diseases and/or conditions associated with AIDS at diagnosis), and ARV-related variables (date of treatment onset, treatment regimen, and changes in therapeutic regimens).

The cohort database was created using the information available in SINAN, later complemented by linkage with the SISCEL (viral load and CD4), SICLOM (treatment regimens) and SIM (death identification) databases. The probabilistic linkage method described by Pires et al. (2011) [20] was employed using the patient’s name, sex, date of birth, mother’s name, municipality of residence, home address and number, and national health registry as variables. When necessary, dubious matches were reevaluated by three reviewers to ensure reliability of the linkage. Once the database used in this study was created, duplications were eliminated and inconsistencies were checked. The data were analyzed using the SPSS 20.0 and STATA 14 software.

### Data analysis

The main characteristics of the study population were described. Categorical variables were compared by the Chi-squared test (*Χ*^2^) and continuous variables by the Student t-test or Mann–Whitney–Wilcoxon test. Data from each of the four regions were analyzed: (i) São Paulo City (capital); (ii) Greater São Paulo Region, excluding the capital (GSPR); (iii) São Paulo State Coastal Area (SPCA), and (iv) Interior São Paulo State (ISPS).

Mortality rates were estimated using deaths from AIDS by follow-up time after diagnosis, in years, as the numerator and the total number of person-time at risk for the event in successive years of follow-up (1st, 2nd, 3rd…. until the 12th year of follow-up) as the denominator [21].

The Kaplan–Meier method was applied to estimate the median survival time of patients with AIDS. The Peto log-rank test was used for the comparison of estimated survival curves. The data were censored in three situations: (i) patients who on December 31, 2014, the date of completion of the study, were alive and under follow-up (administrative censoring); (ii) patients who died from a cause other than AIDS; in this case, the data were censored on the day of death; (iii) patients who dropped out of follow-up, i.e., those who had no new records in SISCEL/SICLOM for a period of 1 year or longer; in this case, the data were censored on the day of this last record.

In order to investigate the association between exposures of interest and time to death from AIDS, the Cox proportional hazards model was applied to estimate the unadjusted and adjusted hazard ratio (HR), with 95% confidence intervals (95% CI). Variables with p < 0.25 in bivariate analysis and/or that exhibited biological plausibility, adjusted for age, sex and year of diagnosis, were included in the final Cox model. The likelihood ratio test and Schoenfeld residuals were used to assess the goodness-of-fit of the final model.

The protocol of this study was submitted to and approved by the Ethics Committee on Research Involving Humans of the STD/AIDS Referral and Training Center, São Paulo State Department of Health (Ethical Clearance Certificate: CAAE 53185116.4.0000.5375; 14/03/2016). Informed consent was not required and obtained as it is a retrospective study. All methods were performed in accordance with the relevant guidelines and regulations and ethics committee directions.

## Results

**Fig. 1:**
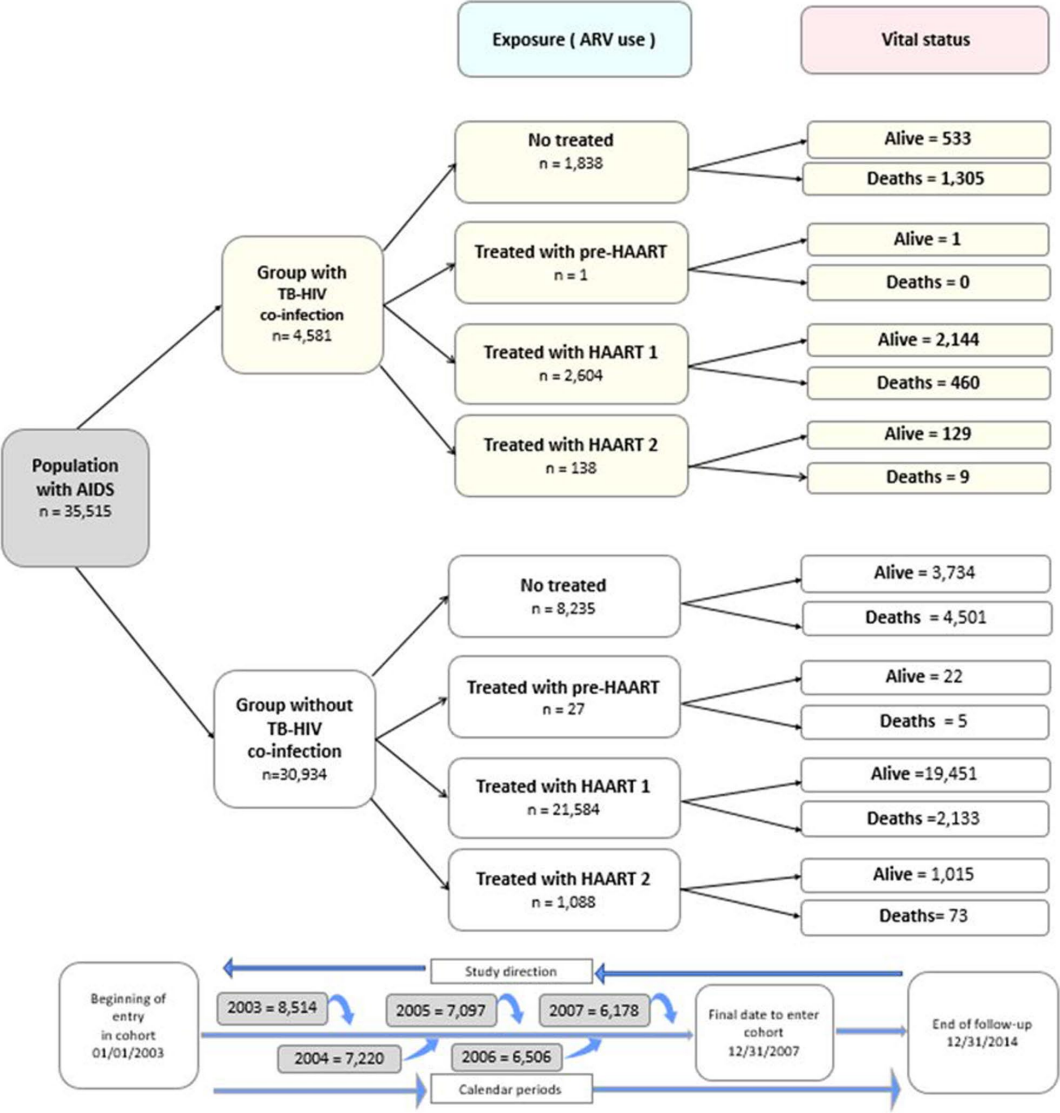
Flow diagram of patients selected for the cohort of patients with AIDS from entry into the study to the end of follow-up according to the presence of TB–HIV co-infection. São Paulo State, 2003 to 2007

Initially, 49,067 new AIDS cases reported to the epidemiological surveillance were identified. According to the inclusion and exclusion criteria of the study, 8278 (16.9%) patients diagnosed with AIDS on the day of death, 4997 (10.2%) who were followed-up for less than 30 days, and 277 (0.6%) with inconsistent data were excluded. The study population consisted of 35,515 patients; of whom, 27,029 (69.1%) were followed-up during the study period, here included 2487 patients who died of other causes than AIDS, and 8486/35,515 (23.9%) who had AIDS as underlying cause of death, totaling 10,973 (30.9%) deaths (Fig. 1). The 2487/35,515 (7.0%) patients who died from causes other than AIDS, mainly cardiovascular diseases and external causes, were not included in the estimated AIDS mortality rates.

There were no differences in age, race/skin color, educational level, risk exposure or region of residence between patients included in the study (35,515) and excluded ones (13,552). However, the prevalence of TB co-infection was higher in the study group than among excluded patients, 12.9% (4581/35,515) versus 7.3% (468/6427), p < 0.001. It should be noted that this information was not available for 7125/13,552 (52.6%) patients.

In the population studied, 63.0% (22,367/35,515) were male, 64.7% (22,963/35,515) belonged to the 30 to 49 years age group, while the mean and median age was 37.4, 95% CI (37.3–37.5) and 36 years, respectively; 64.4% (19,412/30,152) were white (self-reported), 97.2% (33,555/34,530) lived in the urban area, 52.7% (15,261/28,969) had more than 7 years of schooling, 12.9% (4581/35,515) were co-infected with TB, and 37.6% (11,228/29,855) had CD4 count below 200 cells/mm^3^ at AIDS diagnosis (Table 1). The prevalence of TB–HIV co-infection differed among residents in the four regions of the state was: 14.9% (1919/12,880) in the capital, 14.1% (788/5586) in GSPR, 17.5% (365/2085) in SPCA, and 10.1% (1509/14,964) in the ISPS municipalities (p < 0.001).

**Table 1:** Characteristics of patients with AIDS according to TB–HIV co-infection, São Paulo State, 2003 to 2014

Characteristics	TB–HIV co-infection		Total (n = 35,515) N (%)	p-value^+^
	No (n = 30,934) N (%)	Yes (n = 4581) N (%)		
Region of residence				< 0.001
SPC	10,961 (35.4)	1919 (41.9)	12,880 (36.3)	
GSPR^a^	4798 (15.5)	788 (17.2)	5586 (15.7)	
SPCA	1720 (5.6)	365 (8.0)	2085 (5.9)	
ISPS	13,455 (43.5)	1509 (32.9)	14,964 (42.1)	
Sex				< 0.001
Male	18,964 (61.3)	3403 (74.3)	22,367 (63.0)	
Female	11,970 (38.7)	1178 (25.7)	13,148 (37.0)	
Age group (years)				< 0.001
13 to 29	7378 (23.9)	824 (18.0)	8202 (23.1)	
30 to 49	19,735 (63.8)	3228 (70.5)	22,963 (64.7)	
≥ 50	3821 (12.4)	529 (11.5)	4350 (12.2)	
Education (years of schooling)*				< 0.001
Illiterate	577 (2.3)	106 (3.0)	683 (2.4)	
1 to 3	2985 (11.7)	528 (14.8)	3513 (12.1)	
4 to 7	9464 (37.2)	1601 (45.0)	11,065 (38.2)	
8 to 11	9538 (37.5)	1105 (31.1)	10,643 (36.7)	
≥ 12	2849 (11.2)	216 (6.1)	3065 (10.6)	
Race/skin color**				< 0.001
White	17,197 (65.4)	2215 (57.5)	19,412 (64.4)	
Black	6372 (24.2)	1056 (27.4)	7428 (24.6)	
Mixed race/black	2,567 (9.8)	558 (14.5)	3125 (10.4)	
Yellow	129 (0.5)	18 (0.5)	147 (0.5)	
Indigenous	33 (0.1)	7 (0.2)	40 (0.1)	
Area of residence***				0.001
Urban	29,288 (97.3)	4267 (96.5)	33,555 (97.2)	
Rural	658 (2.2)	114 (2.6)	772 (2.2)	
Periurban	161 (0.5)	42 (0.9)	203 (0.6)	
Year of AIDS diagnosis				< 0.001
2003	7246 (23.4)	1268 (27.7)	8514 (24.0)	
2004	6235 (20.2)	985 (21.5)	7220 (20.3)	
2005	6213 (20.1)	884 (19.3)	7097 (20.0)	
2006	5745 (18.6)	761 (16.6)	6506 (18.3)	
2007	5495 (17.8)	683 (14.9)	6178 (17.4)	
Exposure category				< 0.001
MSM	6448 (23.0)	712 (18.2)	7160 (22.4)	
Heterosexual	18,599 (66.4)	2379 (60.9)	20,978 (65.7)	
IDU	2751 (9.8)	788 (20.2)	3539 (11.1)	
CD4 (cells/mm^3^)*****				< 0.001
< 200	9530 (35.9)	1698 (50.8)	11,228 (37.6)	
200–349	10,244 (38.6)	954 (28.5)	11,198 (37.5)	
350–499	3194 (12.0)	339 (10.1)	3533 (11.8)	
≥ 500	3543 (13.4)	353 (10.6)	3896 (13.0)	
Viral load******				< 0.001
≤ 500	3467 (14.2)	512 (16.9)	3979 (14.5)	
> 500	20,864 (85.8)	2517 (83.1)	23,381 (85.5)	
ARV regimen^b^				< 0.001
No ARV	8235 (26.6)	1838 (40.1)	10,073 (28.4)	
Pre-HAART	27 (0.1)	1 (0.0)	28 (0.1)	
HAART1	21,584 (69.8)	2604 (56.8)	24,188 (68.1)	
HAART2	1088 (3.5)	138 (3.0)	1226 (3.5)	

MSM: Men who have sex with men, IDU: Injection drug user; SPC: São Paulo City (Capital); GSPR: Greater São Paulo Region, excluding the capital; SPCA: São Paulo State Coastal Area; ISSP: Interior São Paulo State

Total number of AIDS records ignored for the variable: *6546; **4636; ***827; ****2926; *****4423; ******6603

Total number of TB–HIV records ignored for the variable: *1025; **727; ***158; ****677; *****1237; ******1552

^+^Pearson’s Chi-squared

^a^Excluding São Paulo City

^b^Use of the more complex regimen

Comparison of the characteristics of the patients with and without TB co-infection (4581 and 30,934, respectively) showed a higher proportion of men (74.3% versus 61.3%; p < 0.001), individuals with up to 7 years of schooling (62.8% versus 51.2%; p < 0.001), individuals with a higher mean and median age (38.1 and 37 years versus 37.3 and 36 years), blacks and mixed race/black (41.9% versus 34.0%; p < 0.001), injection drug users (IDU) (20.2% versus 9.8%; p < 0.001), patients with CD4 count below 200 cells/mm^3^ at AIDS diagnosis (50.8% versus 35.9%; p < 0.001), and patients who did not receive ARV (40.1% versus 26.6%; p < 0.001) in the co-infected group (Table 1). The proportion of deaths until the end of follow-up was higher among co-infected patients (38.7% versus 21.7%; p < 0.001) (Table 2).

**Table 2:** Factors associated with survival in patients with AIDS followed for up to 10 years after diagnosis, São Paulo State, 2003 to 2014

Characteristics	Total (n = 35,515)	Dead (n = 8,486)	Alive (n = 27,029)	HR_unadjusted_ (95% CI)	HR_adjusted_ (95% CI)
	N (%)	N (%)	N (%)		
Sex					
Female	13,148 (100.0)	2686 (20.4)	10,462 (79.6)	1	1
Male	22,367 (100.0)	5800 (25.9)	16,567 (74.1)	1.37 (1.31–1.45)	**1.26** (1.14–1.39)
Age group (years)					
13 to 29	8202 (100.0)	1584 (19.3)	6618 (80.7)	1	1
30 to 49	22,963 (100.0)	5564 (24.2)	17,399 (75.8)	1.36 (1.28–1.45)	1.14 (1.04–1.26)
≥ 50	4350 (100.0)	1338 (30.8)	3012 (69.2)	1.94 (1.79–2.11)	**1.35** (1.18–1.54)
Year of AIDS diagnosis					
2007	6178 (100.0)	1202 (19.5)	4976 (80.5)	1	1
2006	6506 (100.0)	1394 (21.4)	5112 (78.6)	1.01 (1.01–1.10)	1.02 (1.01–1.02)
2005	7097 (100.0)	1710 (24.1)	5387 (75.9)	1.06 (1.01–1.18)	1.03 (1.03–1.04)
2004	7220 (100.0)	1846 (25.6)	5374 (74.4)	1.09 (1.01–1.18)	1.05 (1.04–1.06)
2003	8514 (100.0)	2334 (27.4)	6180 (72.6)	1.13 (1.04–1.22)	1.10 (1.05–1.12)
Region*					
GSPR^a^	5586 (100.0)	1252 (22.4)	4334 (77.6)	1	1
SPC	12,880 (100.0)	2994 (23.2)	9886 (76.8)	1.05 (1.02–1.12)	1.16 (1.01–1.32)
ISPS	14,958 (100.0)	3752 (25.1)	11,206 (74.9)	1.14 (1.04–1.16)	**1.43** (1.25–1.62)
SPCA	2085 (100.0)	487 (23.4)	1598 (76.6)	1.33 (1.09–1.42)	**1.49** (1.21–1.82)
Education (years of schooling)					
≥ 12	3065 (100.0)	432 (14.1)	2633 (85.9)	1	1
8 to 11	10,643 (100.0)	1980 (18.6)	8663 (81.4)	1.36 (1.22–1.51)	**1.64** (1.35–1.99)
4 to 7	11,065 (100.0)	2929 (26.5)	8136 (73.5)	2.02 (1.82–2.23)	**2.35** (1.93–2.86)
1 to 3	3513 (100.0)	1086 (30.9)	2427 (69.1)	2.38 (2.02–2.81)	**2.41** (1.77–3.30)
Illiterate	683 (100.0)	209 (30.6)	474 (69.4)	2.42 (2.17–2.71)	**2.61** (2.11–3.24)
Race					
White	19,412 (100.0)	4538 (23.4)	14,874 (76.6)	1	1
Mixed race/black	7428 (100.0)	1706 (23.0)	5722 (77.0)	1.29 (1.19–1.40)	1.07 (1.02–1.18)
Black	3125 (100.0)	916 (29.3)	2209 (70.7)	2.11 (1.22–3.64)	**1.27** (1.12–1.45)
Yellow	147 (100.0)	38 (25.9)	109 (74.1)	1.27 (0.90–1.80)	0.47 (0.17–1.25)
Indigenous	40 (100.0)	14 (35.0)	26 (65.0)	0.96 (0.90–1.02)	1.67 (0.54–5.21)
Exposure category					
MSM	7160 (100.0)	1203 (16.8)	5957 (83.2)	1	1
Heterosexual	20,978 (100.0)	4427 (21.1)	16,551 (78.9)	1.30 (1.21–1.39)	1.16 (1.03–1.31)
IDU	3539 (100.0)	1203 (34.0)	2336 (66.0)	2.35 (2.15–2.58)	**1.73** (1.49–2.02)
TB–HIV co-infection					
No	30,934 (100.0)	6712 (21.7)	24,222 (78.3)	1	1
Yes	4581 (100.0)	1774 (38.7)	2807 (61.3)	2.05 (1.94–2.16)	**1.70** (1.49–1.87)
ARV regimen^b^					
HAART2	1226 (100.0)	82 (6.7)	1144 (93.3)	1	1
HAART1	24,188 (100.0)	2593 (10.7)	21,595 (89.3)	1.71 (1.37–2.13)	**1.94** (1.47–2.55)
Pre-HAART	28 (100.0)	5 (17.9)	23 (82.1)	3.07 (1.25–7.58)	**5.35** (4.03–7.10)
No ARV	10,073 (100.0)	5806 (57.6)	4267 (42.4)	17.6 (14.2–21.9)	**8.22** (2.95–22.87)
CD4 (cells/mm^3^)					
≥ 500	3896 (100.0)	294 (7.5)	3602 (92.5)	1	1
350–499	3533 (100.0)	331 (9.4)	3202 (90.6)	1.26 (1.05–1.50)	**1.31** (1.07–1.60)
200–349	11,198 (100.0)	1263 (11.3)	9935 (88.7)	1.65 (1.43–1.89)	**1.47** (1.25–1.74)
< 200	11,228 (100.0)	2000 (17.8)	9228 (82.2)	2.78 (2.43–3.18)	**2.31** (1.97–2.72)
Viral load					
40–500	3978 (100.0)	327 (8.2)	3651 (91.8)	1	1
> 500	23,381 (100.0)	3281 (14.0)	20,100 (86.0)	1.91 (1.68–2.17)	**1.99** (1.72–2.30)

Cox proportional hazards model

Observation: The difference between *n* and the total number of the categories of each variable corresponds to the number of missing information

MSM: Men who have sex with men; IDU: Injection drug user; HR: hazard ratio; 95% CI: 95% confidence interval; MSM: men having sex with men; IDU: injection drug user; ARV: antiretroviral therapy; Pre-HAART: nucleoside reverse transcriptase inhibitors; HAART1: non-nucleoside reverse transcriptase inhibitors/protease inhibitors; HAART2: regimens containing at least one third-line ARV; SPC: São Paulo City (Capital); GSPR: Greater São Paulo Region, excluding the capital; SPCA: São Paulo State Coastal Area; ISSP: Interior São Paulo State

^a^Excluding São Paulo City

^b^The more complex regimen was used

When we compared the 24,542 patients who continued the follow-up until the end of 2014 with the 8486 patients who had died from AIDS as underlying cause, we found among the patients who died a higher proportion of TB co-infected patients (9.8% versus 20.9%; p < 0.001), individuals with up to 7 years of schooling (11.3% versus 21.7%; p < 0.001), individuals with CD4 count below 200 cells/mm^3^ at diagnosis (13.6% versus 20.9%; p < 0.001), and patients who did not receive ARV (10.0% versus 22.5%; p < 0.001).

**Fig. 2:**
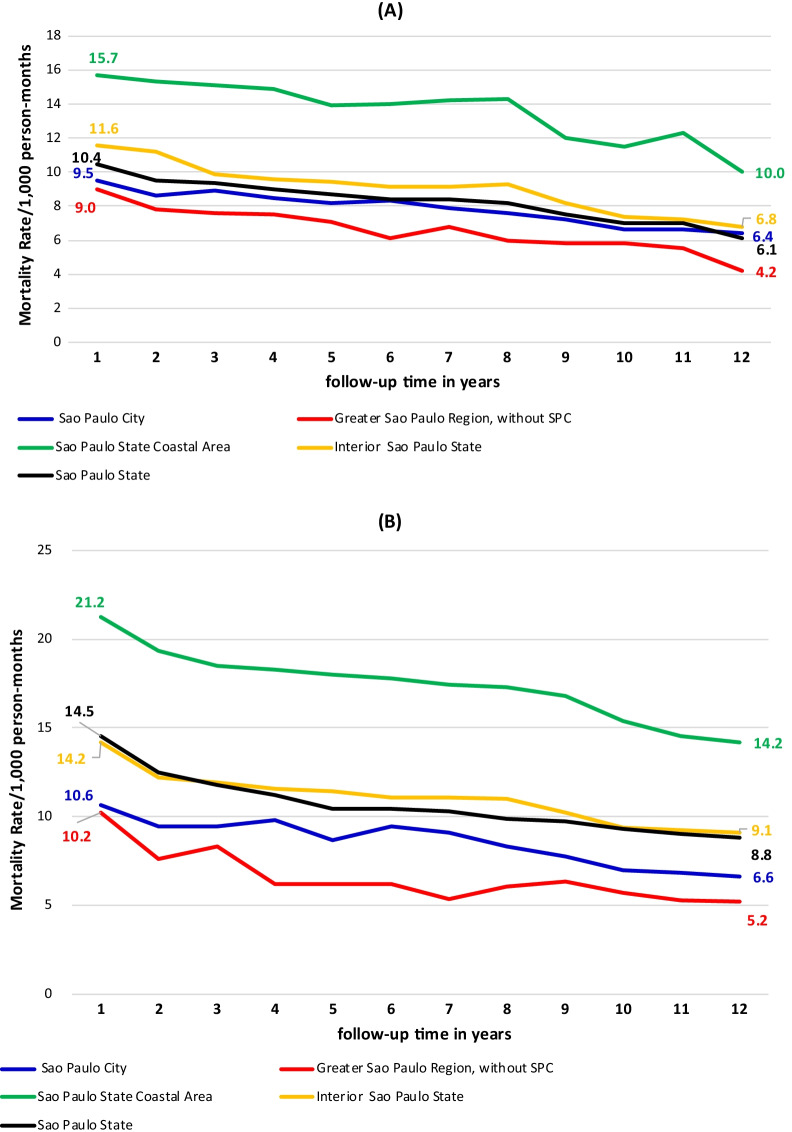
Rate of AIDS mortality* among patients without (**A**) and with TB–HIV co-infection (**B**) according to follow-up time** and region. São Paulo State. *Mortality from AIDS as underlying cause per 1000 person-months. **Follow-up time in years

The AIDS-related mortality rate in the groups with and without TB co-infection was higher in the 1st year after diagnosis and declined progressively until the end of follow-up. However, these rates were not homogenous across the four regions of the state, being expressively higher in SPCA and lower in GSPR. Among non-coinfected patients, respectively comparing SPCA and GSPR residents, the relative risk (RR) was 1.74 (p < 0.001) in the first year of follow-up and 2.38 (p < 0.001) at the end of follow-up (Fig. 2A). Performing the same comparison among co-infected patients, the RR was 2.08 (p < 0.001) in the first year and 2.73 (p < 0.001) at the end of follow-up (Fig. 2B). When the mortality rates at the beginning and end of follow-up were compared between co-infected residents in SPCA and non-coinfected residents in GSPR, the RR was 2.3 and 3.4, respectively (p < 0.001).

**Fig. 3:**
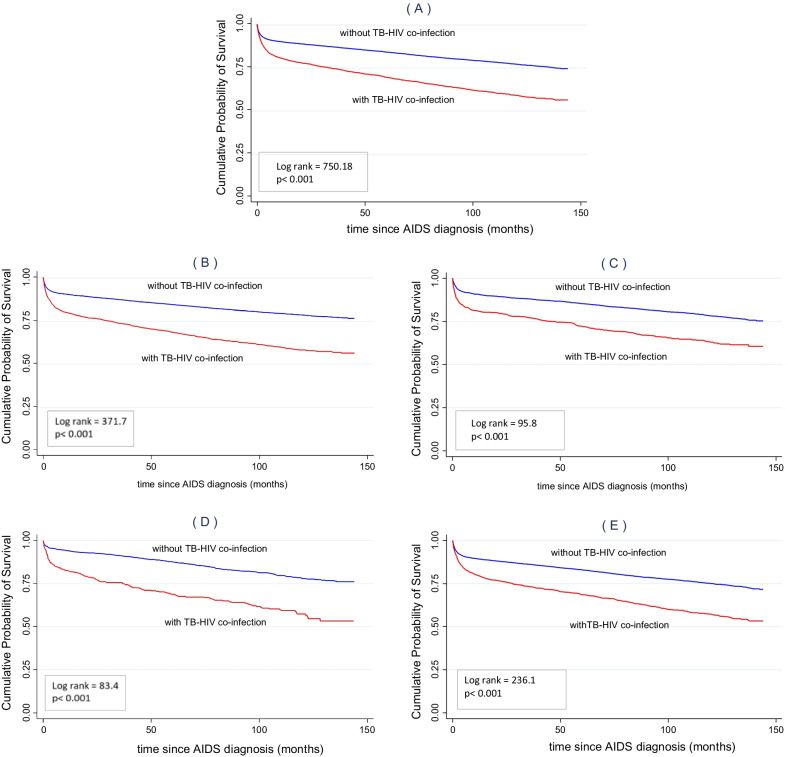
Cumulative probability of survival after the diagnosis of AIDS in cohorts of patients diagnosed between 2003 and 2007 and followed up until 2014 according to the different regions of São Paulo State and the presence or absence of TB–HIV co-infection. Kaplan–Meier curves: **A** São Paulo State, **B** São Paulo City, **C** Greater São Paulo Region, **D** São Paulo State Coastal Area, and **E** Interior São Paulo State

The cumulative probability of survival in the study population was 70.0% at 12 years after diagnosis (144 months) (Fig. 3); however, for co-infected patients, the survival probability was 55.7%, against 74.1% for patients without TB (log-rank = 750.18; p < 0.001) (Fig. 3).

The 12-year survival after AIDS diagnosis in patients without and with TB co-infection was different in the regions of the state, as follows: SPCA: 75.3% versus 60.5% (log-rank = 83.4; p < 0.001); capital: 76.3% versus 55.9% (log-rank = 371.7; p < 0.001); GSPR: 75.9% versus 53.2% (log-rank = 95.8; p < 0.001); ISPS: 71.6% versus 53.2% (log-rank = 236.1; p < 0.001), with significantly lower survival in co-infected patients from all regions (Fig. 3).

**Fig. 4:**
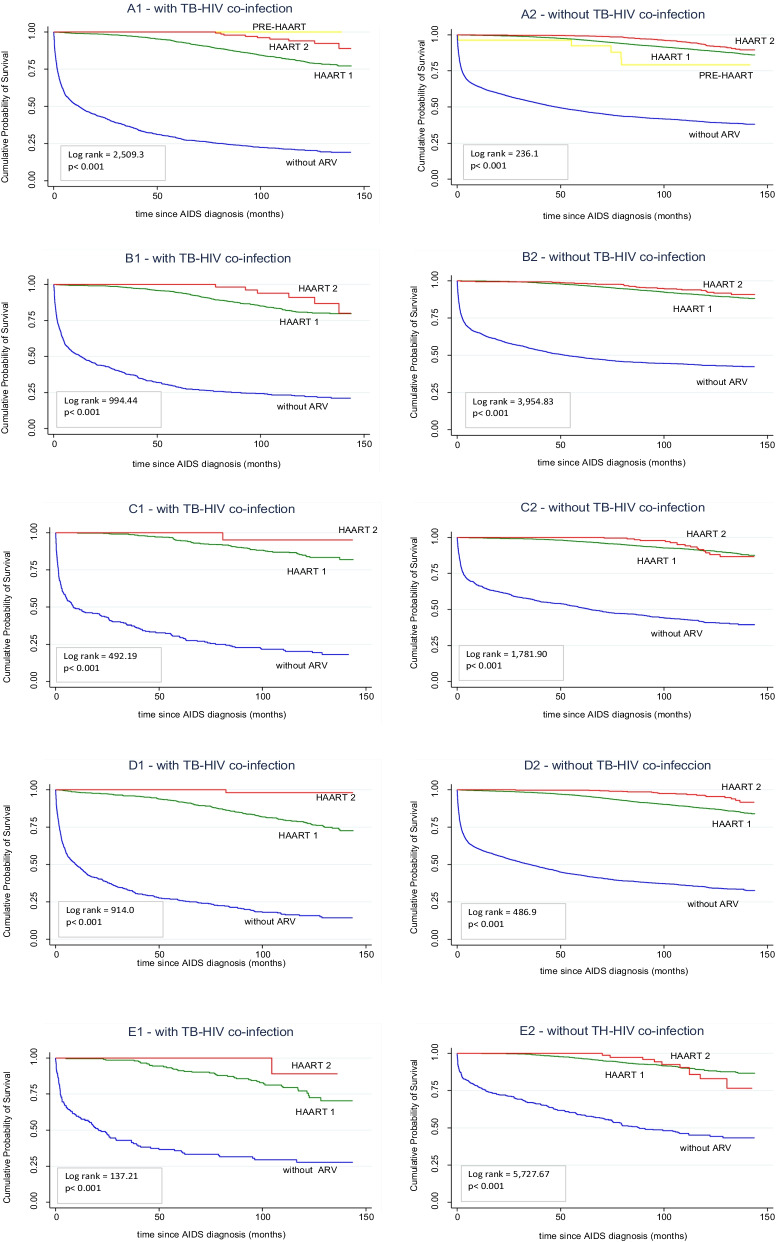
Cumulative probability of survival after the diagnosis of AIDS in cohorts of patients diagnosed between 2003 and 2007 and followed up until 2014 according to the different regions of São Paulo State, the presence or absence of TB–HIV co-infection, and treatment regimen. Kaplan–Meier curves: **A1** and **A2** São Paulo State, **B1** and **B2** São Paulo City, **C1** and **C2** Greater São Paulo Region, **D1** and **D2** São Paulo State Coastal Area, and **E1** and **E2** Interior São Paulo State

Differences in the 12-year survival of patients with and without TB were also observed according to ARV (Fig. 4). In co-infected patients, the 12-year survival probability after diagnosis was 95.2%, 82.9% and 21.9% for HAART2, HAART1 and no ARV, respectively. The SPCA was the region with the lowest survival (88.0%, 70.0% and 27.0%) (Fig. 4). In non-coinfected patients, the 12-year survival probability after diagnosis was 95.2%, 90.5% and 40.9% for HAART2, HAART1 and no ARV, respectively. Among these patients, the SPCA region also showed the lowest survival probabilities (86.6%, 76.5% and 43.4%) (Fig. 4).

The results of bivariate analysis of factors associated with survival are shown in Table 2. In the Cox proportional hazards model, the following exposures were associated with survival time after adjustment for sex, age and year of diagnosis, irrespective of the other variables: area of residence ISPS municipalities (HR = 1.43; 95% CI 1.25–1.62); SPCA (HR = 1.49; 95% CI 1.21–1.82); TB co-infection (HR = 1.70; 95% CI 1.49–1.87); male sex (HR = 1.26; 95% CI 1.14–1.39); being 50 years or older (HR = 1.35; 95% CI 1.18–1.54); black skin color (HR = 1.27; 95% CI 1.12–1.45); 8 to 11 years of schooling (HR = 1.64; 95% CI 1.35–1.99); 4 to 7 years of schooling (HR = 2.35; 95% CI 1.93–2.86); 1 to 3 years of schooling (HR = 2.41; 95% CI 1.77–3.30); illiteracy (HR = 2.61; 95% CI 2.11–3.24); IDU exposure category (HR = 1.73; 95% CI 1.49–2.02); CD4 below 200 cells/mm^3^ at diagnosis (HR = 2.31; 95% CI 1.97–2.72); viral load above 500 copies/ml at diagnosis (HR = 1.99; 95% CI 1.72–2.30); HAART1 (HR = 1.94; 95% CI 1.47–2.55); pre-HAART (HR = 5.35; 95% CI 4.03–7.10), and no ARV use (HR = 8.22; 95% CI 2.95–22.87) (Table 2).

## Discussion

This study comprised four successive annual cohorts of new AIDS cases reported in São Paulo state who were followed up for up to 12 years. The main finding was the heterogeneous mortality and survival among the four state regions, each of them with different AIDS and TB incidences and overall TB mortality. Also, consistent with the literature [8, 22], our results show that mortality is higher in the first year after AIDS diagnosis, and although survival rates are lower for AIDS patients who had TB, wisely prescribed antiretrovirals (ARV) shift this tendency for better [5, 6, 23].

While a universal and free healthcare system with capillarity throughout the territory [23] is in place, a quarter of new AIDS cases was censored because they either died or were lost to follow-up within 30 days of diagnosis. It is of concern that the country policy of free of charge ARV since 1997, and to be prescribed to all HIV infected persons regardless the CD4 count since 2013 [24] did not result in equitable outcomes in the highest HDI scoring state.

Approximately one quarter of the patients followed by the AIDS control program did not receive ARV and account for a high proportion of deaths. This catastrophic finding corroborates the idea that a nationwide treatment policy is not sufficient. While this may reflect programmatic problems, adherence issues in specific populations, such as IDUs, TB co-infected patients, for whom adverse events associated with the dual treatment regimen may pose and extra burden [8] demand specific actions. Late diagnosis, inferred by the high proportion of patients with CD4 below 200 cells/mm^3^ at diagnosis [7] rise concerns regarding access to health care and conditions to be retained in care.

Mortality rates were lower in the GSPR and capital than in the coastal area or interior of the state. A fifth of São Paulo state citizens live in the GSPR in what comprises 3% of the surface area of the state. In that region, AIDS incidence (14.1/100,000) was, in 2014, the lowest of the four regions, and AIDS incidence (30.9/100,000) was only above the one in ISPS, where it was estimated in 21.9. In this setting, mortality rate for TB coinfected patients 1 year after AIDS diagnosis (10.2) was lower than the 14.2 at the end of 12 years follow-up for the same group of patients living in SPCA, and would still reach a low 5.2 in the same period. Along the coast, TB incidence in 2014 was nearly four times that of GSPR, whereas AIDS incidence was 1.6 times higher [9].

A nationwide evaluation classified Brazilian municipalities in lower or higher socio-economic scenarios (LSS and HSS) defined by multiple variables including Gini index, HDI, illiteracy, poverty, among others. LSS municipalities had higher TB incidence and mortality. The whole SPCA was classified in the LSS, whereas part of GSPR, the capital and apart from small islets, ISPS were HSS [25]. This data is consistent with our findings, suggesting that on top of the factors that were negatively associated with survival: older treatment regimens, late AIDS diagnosis, IDU, older than 50 years, lower educational level, TB co-infection, no or inadequate ARV; other social determinants may represent an important component of AIDS patients survival.

Successful prevention strategies and timely effective treatment strategies would probably impact both infections burden and related mortality. Understanding the differences in each of the regions and its specific needs is urgently needed.

The interpretation of the present results must take into account some limitations of the study, including the use of secondary surveillance data that are prone to underreporting and lack of completeness. Data on the treatment regimens refer to the drugs distributed to patients and not necessarily to their effective use. Date of TB occurrence and its treatment outcomes were not included in the analysis and within this context, it is possible that TB timing and unfavorable outcomes, such as TB losses to follow up, may also be associated with mortality [2].

## Conclusions

The research shows that TB co-infection still has a high impact on mortality of patients living with AIDS, with the highest number of deaths occurring in the 1st year after AIDS diagnosis. Within the four São Paulo state regions, mortality rates were worse in the coastal area, where incidence of both infections and social economic indicators are worse. Whether one cause or consequence of the other, it seems that without addressing social disparities, HIV and TB control will not be achieved. The implications of factors associated with survival, with emphasis on age, education, early diagnosis and effective treatment regimens reaffirm this understanding. The richest state of a middle-income country with universal free of charge medical care has yet to find and implement interventions and intersectoral policies to promote health and living equity to patch the gap that still leaves out the most vulnerable.

## Data Availability

The data that support the findings of this study are available from the Brazilian Ministry of Health, but restrictions apply to the availability of these data, which were used under license for the current study, and so are not publicly available. Data are however available from the authors upon reasonable request and with permission of the Ministry of Health.
